# Quality Control of Bacterial Extracellular Vesicles with Total Protein Content Assay, Nanoparticles Tracking Analysis, and Capillary Electrophoresis

**DOI:** 10.3390/ijms23084347

**Published:** 2022-04-14

**Authors:** Aleksandra Steć, Joanna Jońca, Krzysztof Waleron, Małgorzata Waleron, Agata Płoska, Leszek Kalinowski, Bartosz Wielgomas, Szymon Dziomba

**Affiliations:** 1 Department of Toxicology, Faculty of Pharmacy, Medical University of Gdansk, 107 Hallera Street, 80-416 Gdansk, Poland; aleksandra.stec@gumed.edu.pl (A.S.); bartosz.wielgomas@gumed.edu.pl (B.W.); 2 Department of Pharmaceutical Microbiology, Faculty of Pharmacy, Medical University of Gdansk, 107 Hallera Street, 80-416 Gdansk, Poland; joanna.jonca@gumed.edu.pl (J.J.); krzysztof.waleron@gumed.edu.pl (K.W.); 3 Laboratory of Plant Protection and Biotechnology, Intercollegiate Faculty of Biotechnology UG and MUG, 58 Abrahama Street, 80-307 Gdansk, Poland; malgorzata.waleron@biotech.ug.edu.pl; 4 Department of Medical Laboratory Diagnostics—Fahrenheit Biobank BBMRI.pl, Faculty of Pharmacy, Medical University of Gdansk, 7 Debinki Street, 80-211 Gdansk, Poland; szeffler@gumed.edu.pl (A.P.); leszek.kalinowski@gumed.edu.pl (L.K.); 5 BioTechMed Centre, Department of Mechanics of Materials and Structures, Gdansk University of Technology, Narutowicza Street 11/12, 80-233 Gdansk, Poland

**Keywords:** electrophoresis, extracellular vesicles, *Pectobacterium*, purity, subpopulations, ultracentrifugation

## Abstract

Extracellular vesicles (EVs) were isolated from *Pectobacterium zantedeschiae* culturing media using direct ultracentrifugation (UC), iodixanol cushion ultracentrifugation (ICUC), and iodixanol density gradient ultracentrifugation (IDGUC) techniques. The isolates were characterized with total protein content assay (bicinchoninic acid assay, BCA), nanoparticles tracking analysis (NTA), and capillary electrophoresis (CE). A satisfactory correlation (R^2^ > 0.94) between quantitative results obtained with BCA, NTA and CE was achieved only for isolates obtained with the IDGUC. The correlation between protein content and CE was proved to be related to the isolates’ purity. The NTA was found unable to provide reliable information on EVs quantity in samples isolated with UC and ICUC, due to the co-isolated particulate impurities. Moreover, the work reports polysaccharides, used as culturing media components, as a potential source of bias of quantitation with total protein content assay and NTA. The study demonstrates the advantageous selectivity of CE in quality control of EVs and its ability to differentiate subpopulations of EVs of *Pectobacterium*.

## 1. Introduction

Extracellular vesicles (EVs) are spherical nanostructures released by living cells, featuring the ability to transport proteins, lipids, nucleic acids, and metabolites that determine their biological activity [1,2]. EVs are exploited by bacteria as genes and as virulence factors transporters, nutrition scavengers, and decoys against pathogens such as phages [3]. Secreted from the surface of the cell membrane, EVs carry a whole spectrum of antigens typical for certain bacteria strains that found application in immunology and vaccines production [4]. The eukaryotic vesicles are also investigated as drug delivery systems [5] and diagnostic targets [6]. While these examples describe only a small part of the role and utility of EVs, the great interest gained in recent years by this topic is justified.

As biological structures, EVs are liable to physical factors and chemicals to which they are exposed during the isolation process. Some isolation techniques have been shown to affect the integrity and activity of EVs. The choice of isolation protocol is also critical in terms of the purity of EVs [1,2,7,8]. This makes the strict quality control of isolates an inherent part of all research in this field that consumes a significant part of the funds and time of every project. According to the International Society for Extracellular Vesicles (ISEV) latest recommendations, quantitation of EVs should be based on the combination of total macro components (such as proteins or lipids) content assays, and particle counting techniques (typically nanoparticles tracking analysis, NTA, or tunable resistive pulse sensing, TRPS). Moreover, the ratios of certain macro components to the others should be reported as a measure of purity [9]. However, neither macro components content nor particles number is specific for EVs. The presence of soluble (such as non-vesicle proteins, e.g., albumins) and insoluble impurities (lipoproteins, viruses, aggregates of damaged vesicles, cell components, and others) was reported to affect EVs quantitation reliability. Thus, insufficient purity of investigated isolates might be found problematic for the techniques recommended by ISEV, due to their inadequate selectivity [1,2,7,8]. The attention should be paid to the fact that achievement of excellent quality of isolates is not always feasible, as it might be restricted by costs, time, and availability of sample amount [10]. On the other hand, the selectivity of currently used techniques does not meet the stringent criteria set for pharmaceutics and does not correspond to the needs arising from the medical use of EVs (https://database.ich.org/sites/default/files/Q6B_Guideline.pdf; accessed on 20 October 2021).

Characterization of EVs with capillary electrophoresis (CE) has initially been presented on-chip [11]. Application of dark field microscopy enabled single vesicle detection in the microfluidic channel, which was used for zeta potential estimation of EVs in investigated samples [11,12,13,14]. It was also shown that the instrument provides quantitative information [15]. Lan and the group were the first to report the use of the CE in EVs isolates analysis. The team observed differences in electrophoretic mobility between two fractions of EVs isolated from human urine samples with density gradient ultracentrifugation (UC) [16]. Our group explored the CE as a tool for bacterial EVs characterization. It was shown that the use of simple UV detection is sufficient for quantification of EVs and detection of macromolecular aggregates presence in isolates [17,18]. *Morani* and coworkers demonstrated the application of CE with laser-induced fluorescence detection (LIF) for the characterization of human and bovine vesicles stained with fluorescein derivative [19]. *Tani* and *Kaneta* developed an indirect method for the quantitation of EVs. It was based on the determination of fluorescent anti-CD-63 antibodies retained by the vesicles during off-line incubation [20]. *Berezovski’s* group focused on nucleic acids being a part of EVs cargo. The CE-LIF method enabled quantitation of RNA released from vesicles and indirect determination of EVs content in isolates obtained from cells cultures media. The authors pointed out that their method is not able to distinguish populations of EVs and requires knowledge on average RNA content in certain vesicles [21]. In our recent work, we have shown that the CE is suitable for the detection of EVs that transport green fluorescent protein (GFP). The application of UV and LIF detector enabled us to distinguish vesicles secreted by wild-type and GFP-producing bacterial strains [22]. On the one hand, the application of fluorescent probes in the referred works provided high selectivity towards EVs. On the other hand, CE-LIF analysis of such samples does not provide information on other components of the isolate, such as impurities [19,20,21,22].

In the present work, we demonstrate the application of CE for quantitation of EVs obtained from *Pectobacterium* sp. culturing media using direct UC, iodixanol cushion UC (ICUC), and iodixanol density gradient UC (IDGUC). The quality of isolates and their impact on quantification of EVs with CE, total protein content assay (bicinchoninic acid test, BCA), and NTA is discussed. We show that quantitation of EVs with CE is less affected by the presence of impurities, among which we identified polysaccharides used for bacteria cultivation. The ability of CE to separate bacterial subpopulations of EVs is also presented.

## 2. Results

### 2.1. Isolation Protocol

**Figure 1:**
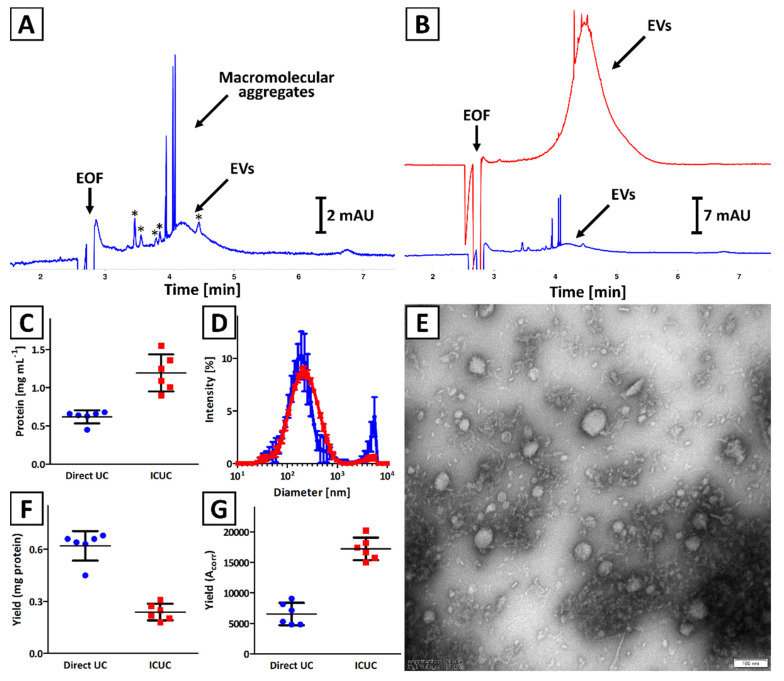
Comparison of isolates obtained with (blue trace) direct UC and (red trace) ICUC methods. (**A**,**B**) CE analysis of exemplary isolates. Asterisks (*) indicate soluble impurities. (**C**) Total protein concentration was assessed with a BCA test. (**D**) DLS analysis of isolates. Error bars indicate the standard deviation (SD) of measurements performed for 3 independently obtained isolates. (**E**) TEM image of an isolate obtained with ICUC technique. The size bar is equal to 100 nm. (**F**) Isolation yield is expressed as total protein content. (**G**) Isolation yield calculated based on CE analyses results (A_corr_) using Equation (2). Bars (**C**,**F**,**G**) represent the mean and SD calculated for 6 independently obtained isolates.

The quality of isolates obtained with direct UC and ICUC methods was compared. The CE results indicated a significantly higher concentration of EVs in isolates obtained with ICUC methodology as compared to direct UC (Figure 1A,B), which was confirmed with the BCA test (Figure 1C). The presence of macromolecular aggregates in isolates obtained with both methodologies was observed in CE (Figure 1B), which was confirmed with DLS analysis (presence of particles with diameter >1 µm; Figure 1D). The polydispersity of isolates obtained with direct UC was also found to be greater (polydispersity index: 0.61 ± 0.05 vs. 0.32 ± 0.03, respectively). Moreover, the CE analysis showed that soluble impurities (indicated with asterisks in Figure 1A) were more abundant in direct UC isolates (Figure 1B). TEM analysis confirmed the presence of spherical nanostructures and some minor solid impurities in isolates obtained with direct UC and ICUC methods (Figure S1 and Figure 1E, respectively).

Significant differences were observed in isolation yield when BCA and CE results were compared (Figure 1F,G). According to the BCA test, a greater amount of EVs was isolated with direct UC, while the CE results suggest that more EVs were obtained with ICUC method. The differences in quality of isolates obtained with direct UC and ICUC were considered to be responsible for this contradiction, which is discussed in Section 4.

The isolates were characterized with NTA (Figure S2). NTA characteristics of isolates, described by various central tendency measures, are presented in Figure S3A–C. Overall, no statistical (Kruskal–Wallis test) differences were observed between Direct UC and ICUC, regardless of the method of expressing the central tendency measurement (mean, mode or median). Statistically significant differences were observed between the ratios of particles number per µg of protein determined for isolates obtained with both methodologies.

### 2.2. Quantification of EVs

The linear correlation (R^2^ = 0.97) between the BCA test and CE analysis was found in a range of 0.1–4.2 mg mL^−1^ of total protein concentration for the isolates obtained with ICUC method. The presence of impurities as (discussed in Section 2.1) justifies the imperfect fit to linear regression. The intra- and inter-day repeatability, determined for the lower limit of quantification (protein concentration: 0.1 mg mL^−1^; signal to noise ratio about 10), was 4.4 (determined for six repeatable measurements) and 9.0% (determined for three measurements performed for 3 consecutive days), respectively. These results were found typical for CE injection repeatability for the lower limit of quantification level [23]. The signals generated by the vesicles were clearly distinguishable from the baseline even at total protein concentration in isolates equal to 0.05 mg mL^−1^ (signal to noise ratio about 5; limit of detection).

The CE method enabled simultaneous monitoring of the residue of iodixanol that was used in the isolation process. The method showed a linear response between iodixanol’s content and A_corr_ in the concentration range from 0.005 to 0.1% (*w*/*v*). As low as 0.001% iodixanol concentration could be detected. Due to the lack of dissociating moieties, iodixanol was migrating with the electroosmotic flow velocity. However, its detection and quantification were possible due to the selective absorbance maximum at 246 nm, which also allowed for spectral purity assessment of the signal.

**Figure 2:**
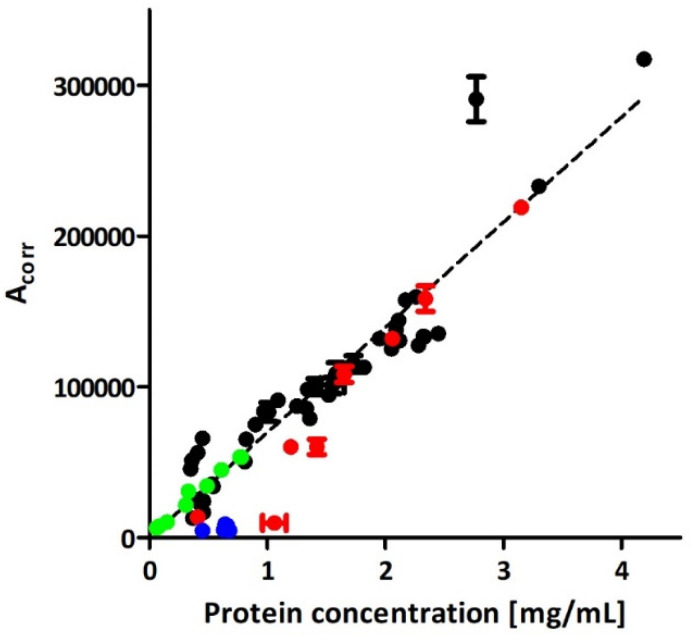
Comparison of EVs quantitation with CE and BCA in isolates obtained with direct UC (blue trace), ICUC (black and red traces), and IDGUC (green trace) methods. The red trace represents isolates that, based on the CE analysis, were considered impure (comment in Section 3). The linear curve was plotted based on the quantitative data obtained for 42 independent isolates, achieved with the ICUC method (black trace). Error bars represent the SD of two measurements.

No relationship was found between CE and NTA or between BCA and NTA results,. While a substantial correlation was achieved between the CE and BCA assay (Figure 2), the presence of particulate impurities (Section 2.1) interfering with EVs in NTA analysis was assumed.

### 2.3. Negative Control

**Figure 3:**
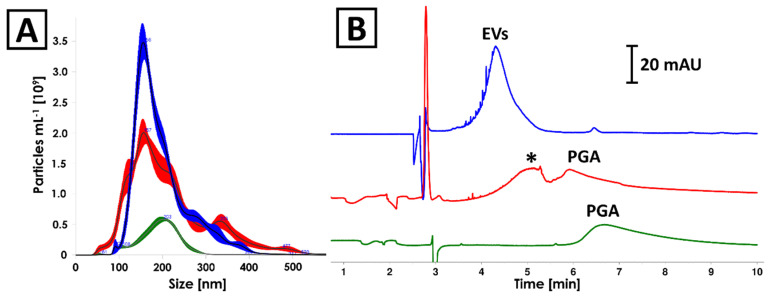
Comparison of (**A**) NTA and (**B**) CE analyses results of isolates obtained from (blue) *Pectobacterium zantedeschiae* culture, (red) culturing medium composed of M63 medium, 0.2% glycerol and 0.4% PGA, and (green) 0.4% PGA solution. The isolation process was described in Section 4.6. Asterisk (*) indicates unidentified component of the culturing medium isolate.

The culturing medium as well as 0.4% PGA solution (the component of culturing medium) have undergone an isolation process according to the ICUC methodology described in Section 4.6. The protein content in the isolates obtained from culturing medium and 0.4% PGA solution was determined on a relatively high concentration level (about 2.6 and 0.9 mg mL^−1^, respectively). The NTA analyses revealed the presence of nanoparticles featuring similar size distribution to EVs produced by *Pectobacterium zantedeschiae* (Figure 3A). The same samples were analyzed with CE, which showed signals characteristic for nanoparticles (efficiency <20,000 plates m^−1^; Figure 3B) in all analyzed isolates [24]. PGA was identified as a component of culturing medium isolate (Figure 3B, red and green trace). Despite the identity of the second component (marked with asterisk in Figure 3B) not being confirmed, the differences in electrophoretic mobility of detected compounds enabled their differentiation from EVs. None of the signals detected in negative control samples was found in isolates of *Pectobacterium* EVs (Figure 3B). Finally, TEM analysis confirmed the absence of vesicles in isolates obtained from culturing media (Figure S4).

### 2.4. Iodixanol Density Gradient UC

The IDGUC technique was applied for EVs isolation from *Pectobacterium odoriferum* Car1 culturing medium. The strain was modified with pPROBE-AT-gfp plasmid to enhance EVs secretion by the cells and to increase the sensitivity of the method [22].

**Figure 4:**
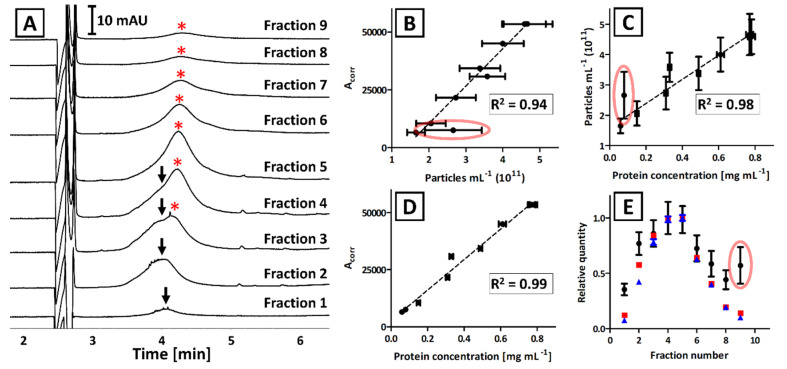
The analysis results of isolates that were obtained from GFP-modified *Pectobacterium odoriferum* Car1 with IDGUC. (**A**) CE analysis of certain fractions. Arrows and asterisks (*) indicate signals generated by two subpopulations of EVs. (**B**–**E**) Comparison of CE, NTA, and BCA analyses of separated fractions. Error bars represent the standard deviation of measurements. Coefficients of determination (R^2^) refer to the linear correlation between certain analyses and do not include the outlier (circled in red). (**E**) The traces correspond to the relative quantity of EVs determined with CE (red), BCA (blue), and NTA (black) in certain fractions.

Each fraction obtained with the IDGUC was characterized with CE, BCA, and NTA (Figure 4A–D). Linear correlation between CE and BCA measurements (R^2^ = 0.99; Figure 4D) was superior over the results that included NTA analyses (R^2^ = 0.93 and 0.89 for Figure 4B,C, respectively). The correlation between NTA results with CE and BCA was significantly greater when outlier (circled in red, Figure 4B,C) was excluded (R^2^ = 0.94 and 0.98, respectively).

The EVs obtained with the IDGUC technique were found larger when compared to UC or ICUC (Figure S3A–C). However, no significant dissimilarities in the size distribution of EVs were observed between collected fractions (mean: 143 ± 6 nm; mode: 125 ± 15 nm; median: 135 ± 6 nm; Figure S5).

The CE analyses revealed the presence of two subpopulations of EVs featuring nonidentical electrophoretic mobility (Figure 4A). This subtle difference was not noticeable in bulk isolates obtained with direct UC and ICUC, due to the insufficient resolution of the CE method (Figure 1). Despite numerous attempts being made (modification of capillary length, BGE composition, and ionic strength), we were not successful in the electrophoretic separation of these subpopulations in isolates obtained with direct UC and ICUC. However, the CE analysis of IDGUC fractionations proved the diversity of isolated EVs.

## 3. Discussion

The UC is the most often used technique for the isolation of EVs from culturing media [25]. Its implementation enables the concentration of vesicles in samples ranging from a few to even hundreds of mL. While the isolation process is relatively uncomplicated and cost effective, the centrifugal force used for sedimentation can induce aggregation of EVs and membrane integrity disruption [26]. These alterations were reported to affect the biological properties of EVs [27]. Our group has recently demonstrated that the CE can distinguish EVs from macromolecular aggregates that were formed during direct UC [17,18]. The aggregates were observed in electropherograms in a form of highly efficient (>1 mln plates m^−^^1^) signals, which in the literature are often referred to as spikes [28,29]. Due to their irregular size and shape, these structures do not feature any defined electrophoretic mobility, and their detection with UV detector results from the light scattering. Certainly, the presence of macromolecular aggregates is undesirable and affects EVs quantitation with typically used protein assays and NTA analysis [1,7].

In the present study, the ICUC technique was used to minimize vesicles damage, aggregation, and to improve the yield and purity of isolates [30,31]. Both BCA and CE confirmed that application of ICUC methodology provided more concentrated isolates. The samples acquired with the ICUC method were devoid of significant amounts of soluble impurities, which were abundant in isolates obtained with direct UC (indicated with asterisks in Figure 1A), and were characterized by the lower number and less intense ‘spikes’, which demonstrated their improved quality (red trace in Figure 1B). The latter observation is in line with DLS results. The ratio of intensities of signals generated by micro- and nanoparticles in samples acquired with direct UC was more than six-fold greater as compared to isolates achieved with the ICUC method. It was also reflected in greater polydispersity of the isolates obtained with direct UC (polydispersity index: 0.61 ± 0.05 vs. 0.32 ± 0.03, respectively).

In our proof of concept work, insufficient purity of isolates accounted for a poor linear correlation between total protein content in isolates and the corrected peak area of EVs in CE (R^2^ = 0.81) [17]. The application of iodixanol cushion enabled significant improvement of the quality of isolates, which is reflected in the superior correlation between the CE and protein assay results (R^2^ = 0.97; Figure 2). Despite the iodixanol did not completely eliminating mechanical stress generated during centrifugation, the purity of ICUC isolates was considered superior over the isolates obtained with direct UC which explain the differences in isolation yields assessed with BCA and CE analyses (Figure 1F,G). The results indicate the overestimation of EVs content in direct UC samples with BCA due to the presence of abundant soluble and insoluble impurities. Indeed, direct UC isolates featured significantly higher content of proteins than expected from the calibration plot obtained for ICUC samples. A similar tendency was observed for the ICUC isolates, whose quality was considered low and were low abundant in EVs (red trace in Figure 2). The CE analysis of these low-purity isolates showed several additional signals (Figure S6), some of which were generated by macromolecular aggregates. However, the tendency seems to be less evident in samples with higher EVs content (red trace in Figure 2). No correlation between the isolates’ quality and bacterial strain type was found.

These findings are also confirmed by the significantly greater number of particles per µg of protein in ICUC isolates (Figure S3D) [32]. However, attention should be paid to the fact that NTA is not able to provide complete information on the particle size distribution and concentration in analyzed samples as particles >1 µm are out of the range of the device [33]. Moreover, the BCA and CE quantitative results showed no correlation with NTA when direct UC and ICUC isolates were analyzed.

The correlation between all three techniques was observed for IDGUC isolates (Figure 4). The isolates obtained with density gradient centrifugation are often reported to outperform other techniques in terms of efficiency and purity of EVs [32,34,35,36]. Superior correlation between BCA and CE results as compared to direct UC and ICUC, and high correlation between these techniques and NTA, confirms the advantageous quality of IDGUC isolates. The correlation between all three techniques is also evident when a relative quantity of EVs in each fraction is compared (Figure 4E). According to Figure 4E, the outlier was observed with the NTA measurement of the last fraction, which can be explained with the co-isolation of particulate impurities. TEM analysis of the 9th fraction did not identify the impurity (Figure S7) and no significant differences between fractions were observed in vesicles’ size distribution (NTA analysis). We hypothesized that the quantification bias in NTA (Figure 4) might be due to the bacterial flagella that could be found on some TEM images of the 9th fraction (Figure S7). Similar structures were observed, e.g., in TEM images of isolates obtained from *Pectobacterium sp.* culturing media with direct UC [17]. In our recent work we have also identified several flagellar proteins in isolates obtained with the ICUC method [22]. However, the obtained evidence is not sufficient to prove the constructed hypothesis.

The negative control used in the study have indicated culturing medium components as an alternative source of contamination that can led to overestimation of EVs content with BCA and NTA (Section 2.3). The culturing of *Pectobacterium* strains was conducted with the use of M63 medium supplemented with glycerol and PGA (a detailed description can be found in Section 4.6), which served as a carbon source in the culturing medium [37]. In the presented study, the PGA was identified among the components that might be co-isolated with EVs and interfere in BCA and NTA assays. It is due to the formation of nanostructures [38] and reducing activity as to which property is responsible for the reactance in Bradford and BCA tests [39]. Interestingly, the CE was able to distinguish interfering components from EVs. It should be emphasized that none of the signals detected in negative control samples (red trace in Figure 3B) was observed in isolates obtained from *Pectobacterium* cultures in the presented study. This indicates that the significant part of the carbohydrate was digested by bacteria. The impact of PGA on BCA and NTA measurements is expected to be dependent on the time of culturing. In the late logarithmic phase of growth, there is a decrease in PGA concentration in the medium as a result of the activity of pectate lyases secreted by bacteria, as it is the only carbon source, after fast consumed glycerol, in this medium. Nonetheless, polysaccharides used for bacteria culturing should be considered as a potential source of the inaccuracy of quantification of EVs with NTA and chemical tests based on oxidoreductive reactions like BCA.

The application of the CE technique enabled us to observe differences in electrophoretic mobility between EVs isolated with the IDGUC method. The diversity was not detected with TEM, which rules out the hypothesis on the separation of single- and double-membrane EVs [40]. Moreover, the peaks detected in all fractions featured fluorescence, which indicates that both subpopulations were transporting GFP [22]. *Lan* and coworkers have already reported various electrophoretic mobility of EVs separated with sucrose density gradient UC [16]. The variety in electrophoretic mobility of subpopulations can be explained by the capacitive effect, which is based on the difference in electric charge on two sides of the liquid membrane of the particle [41,42,43]. The phenomenon can be observed assuming the differences in the cargo transported by the vesicles. Indeed, the isolation in density gradient centrifugation is based on the differences in separated compounds density and thus composition. While we were not able to verify the differences in the composition of the cargo of separated vesicles, the issue is within the scope of our future research.

## 4. Materials and Methods

### 4.1. Materials

BIS-Tris propane (1,3-Bis[tris(hydroxymethyl)methylamino]propane; BTP), bovine serum albumin (BSA), glycine, sodium dodecylsulfate (SDS) and Tris (2-Amino-2-hydroxymethyl-propane-1,3-diol) were obtained from Merck (Darmstadt, Germany). Sodium hydroxide and 0.1 M HCl solution were purchased from Avantor (Gliwice, Poland). All chemicals were of analytical grade. Water used in experiments was deionized with the Basic 5 system (Hydrolab, Wislina, Poland).

The materials for bacterial media preparation were obtained from Pol-Aura with exception of polygalacturonic acid sodium salt (PGA, from citrus fruit, >75%) and ampicillin sodium salt, which were obtained from Sigma Aldrich (Saint Louis, MO, USA).

### 4.2. Protein Assay Kit

Total protein content measurements were performed with Pierce BCA Protein Assay Kit (Thermo Fisher Scientific, Waltham, MA, USA) according to manufacturer recommendations. Both samples and standards were mixed with 6% SDS in a 9 to 1 ratio before the assay. The assay was performed in 96-well plates using the Infinite M200 plate reader (Tecan, Mannedorf, Switzerland).

### 4.3. Nanoparticles Tracking Analysis

All NTA measurements were performed with an NS300 unit (Malvern Panalytical, Malvern, UK). The instrument was equipped with a 405 nm laser, a high sensitivity sCMOS camera, and a syringe pump. Before analysis, the samples were vortexed and diluted to 1 mL with buffer (20 mM Tris/HCl, pH 7.4) to obtain concentrations in the range 10^7^–10^9^ particles mL^−^^1^ (corresponding to 20–100 particles per frame). During the measurement, five 60 **s** films were recorded at 25 °C and a pump flow of 100 µL min^−^^1^ was used. Nanosight 3.4 software was used for analysis with standard settings and a detection threshold set to 5. The camera level was manually adjusted by the operator for each sample, typically ranging from 15 to 16. The number of recorded tracks was always greater than the minimum proposed by the instrument vendor (1000 tracks) to minimize distortion caused by larger particles [44].

Statistical analyses were performed using GraphPad Prism Version 9.2.0 (GraphPad Software, LLC; San Diego, CA, USA). Multiple comparisons were made using the non-parametric Kruskal–Wallis test and a significance level of 0.05.

### 4.4. Dynamic Light Scattering

DLS measurements were performed with Nanosizer ZS using back-scattering mode (173°) at 25 °C. The refractive index of the material and dispersant were 1.45 (protein) and 1.33 (water), respectively. The viscosity was set at 0.8872 mPa s and material absorption was 0.001. Each sample was measured in triplicate. The experiments were conducted with a low volume quartz glass cuvette (3 × 3 mm light path; Hellma Analytics, Mullheim, Germany).

### 4.5. Capillary Electrophoresis

The experiments were conducted in uncoated fused silica capillaries (50 µm i.d. × 30.2 cm) obtained from Polymicro Technologies (West Yorkshire, UK) using P/ACE MDQ plus system (Sciex, Framingham, MA, USA) at 10 kV constant electric voltage. Both samples and capillary were thermostated at 25 °C. The system was equipped with PDA detector and the analyses were monitored at 200 and 230 nm (the latter wavelength was used for peak identity confirmation) using 32 Karat software (version 10.2; Sciex). In the experiments with laser-induced fluorescence detection (LIF), the excitation and emission wavelengths were set at 488 and 520 nm, respectively.

Background electrolyte (BGE) was composed of 50 mM BTP and 75 mM Gly (pH 9.5). The BGE solution was filtered through the nylon syringe filter (0.2 µm, Avantor) and stored at room temperature for up to two weeks.

Before each run, the capillary was rinsed with 0.1 M solution of NaOH (5 min), water (1 min), and BGE solution (5 min). Before sample injection, the capillary was dipped in water to avoid sample contamination. The injection was performed for 5 s at 3.45 kPa, which was followed by post-injection of BGE under the same conditions. The voltage was applied gradually for 0.5 min until 10 kV was reached. The analysis was conducted for 10 min during which the electric current was on the constant level of about 6–7 µA. Other rinsing procedures were described elsewhere [18].

Corrected area of signals (A_corr_) was used for quantitation, which was calculated with 32 Karat software using the following formula:

A_corr_ = (L_d_ A) t^−1^, (1)

L_d_—capillary length to the detector; A—peak area; t—peak migration time.

### 4.6. Bacteria Culturing and EVs Isolation

Bacteria culturing, modification and isolation were performed according to the procedures described elsewhere [22]. *Pectobacterium zantedeschiae* 9M (PCM2893 = DSM105717 = IFB9009) [45] was obtained from a collection of the Laboratory of Plant Protection and Biotechnology of the Intercollegiate Faculty of Biotechnology University of Gdansk and Medical University of Gdansk. *P. odoriferum* Car1 strain was obtained from the Department of Pharmaceutical Microbiology Medical University of Gdansk collection of strains. GFP-tagged strains, *P. zantedeschiae* 9M GFP, and *P. odoriferum* Car1 GFP were created by introducing into wild-type strains cells of a pPROBE-AT-gfp plasmid [46]. Bacteria were stored in frozen glycerol stocks at −80 °C and maintained on Crystal Violet Pectate (CVP) plates [47]. The agar plates used for the cultivation of GFP-tagged strains contained 200 mg L^−^^1^ of ampicillin.

*P. zantedeschiae* 9M and *P. odoriferum* Car1 were grown on Lysogeny Agar (LA) plates overnight at 28 °C. The GFP-tagged strains were grown on the LA medium supplemented with 200 mg L^−^^1^ ampicillin. Bacterial colonies were scraped off the agar and 0.5 McF suspension was prepared in PBS. M63 medium supplemented with 0.2% glycerol and 0.4% PGA [48] was inoculated with the prepared bacterial suspension. The dilution factor was 1:300. Bacteria were grown to the late logarithmic phase of growth (OD = 0.8) at 28 °C with shaking (100 rpm). Then, two subsequent centrifugation steps at 8000× *g* and 10,000× *g* (7888 and 8819 rpm, respectively; Eppendorf Centrifuge 5804 R, F34-6-38 rotor), each for 10 min at 10 °C, were performed. The supernatant was collected and filtered through 0.45 μm pore size nitrocellulose filter (Millipore). The sterility of the filtrate was confirmed by a standard plate count method.

If a culture volume larger than 50 mL was used for the EVs isolation (200 mL or 2 L), the filtrate was concentrated before the ultracentrifugation in a Vivaspin 20 (PES membrane; 300 kDa molecular weight cut off, MWCO) centrifugal concentrators (Sartorius) at 3000× *g* (4045 rpm; Eppendorf Centrifuge 5804 R) for 2 h at 10 °C. For isolation of the membrane vesicles from the obtained filtrates, three different methods were used: direct UC, ICUC, and IDGUC.

In the case of the direct UC method, 40 mL of the filtrate was ultracentrifuged at 85,000 g (25,000 rpm; Beckman L7-55, SW-28 rotor) for 4 h at 10 °C. The pellet containing membrane vesicles was washed once with 20 mM Tris-HCl buffer solution (pH 7.4) and resuspended in 1000 µL of the same buffer.

In the ICUC method, 35 mL of the filtrate was layered on top of 5 mL 40% iodixanol cushion and centrifuged at 85,000× *g* (25,000 rpm; Beckman L7-55, SW-28 rotor) for 4 **h** at 10 °C. The vesicles were collected from the top of the iodixanol cushion. To remove residual iodixanol, the samples were subsequently subjected to ultrafiltration in Vivaspin 20 (PES membrane; 300 kDa MWCO) columns at 3000× *g* (4045 rpm; Eppendorf Centrifuge 5804 R, S-4-72 rotor) for 3 **h** at 10 °C, with one washing step to remove iodixanol. The samples were diluted in Tris-HCl buffer (pH 7.4) to a final volume of 400 µL.

The membrane vesicles of *P. odoriferum* Car1 GFP strain were subjected to the IDGUC method. Iodixanol was diluted with sterile Tris-HCl buffer (pH 7.4) to the final concentrations of 25, 30, 35, 40, 45, and 50% (*v*/*v*). An amount of 5 mL of each of the iodixanol solutions was layered in an ultracentrifugation tube. The vesicles were diluted in the 20% iodixanol to the final volume of 5 mL and layered on the top of the gradient. The samples were centrifuged at 85,000× *g* (25,000 rpm; Beckman L7-55, SW-28 rotor) for 20 **h** at 10 °C. After centrifugation, the fractions were collected (2 mL each) and subjected to ultrafiltration in Vivaspin 20 (PES membrane; 300 kDa MWCO) columns at 3000× *g* (4045 rpm; Eppendorf Centrifuge 5804 R, S-4-72 rotor) for 3 **h** at 10 °C to remove iodixanol with one washing step. The samples were subsequently diluted in Tris-HCl buffer (pH 7.4), to the final volume of 200 µL, and stored at −20 °C.

The isolation yield was calculated with the following equation:

Yield = C_EVs_ V, (2)

C_EVs_—concentration of EVs (protein amount, particles number, or A_corr_) per certain volume unit; V—final volume of the isolate.

### 4.7. Transmission Electron Microscopy

TEM imaging was performed according to the procedure described in [17]. The isolates (5 µL) were deposed on the formvar support on copper mesh (200 mesh, Agar Scientific, Stansted, UK). After solvent evaporation, the samples were contrasted with a 1% uranyl acetate and left for drying. The preparation was investigated with the use of the Tecnai G2 T12 Spirit BioTwin microscope (FEI Company, Hillsboro, OR, USA).

## 5. Conclusions

The quantification of EVs with CE method, total protein content assay (BCA), and NTA was compared. It was shown that the corrected area of peak generated by EVs in CE corresponds to the protein content and particles number. The correlation between CE and BCA was shown to be greater in isolates of superior quality. Moreover, BCA and NTA were proved to be susceptible to the presence of polycarbohydrates that are used as bacteria culturing media components. This problem was not observed in the case of CE, due to the separation of sample constituents. The selectivity of the CE is also reflected in the ability to separate subpopulations of EVs. At the same time, the CE provides qualitative and quantitative information on contaminants present in isolates. This feature might be found favorable for such applications as quality control of biotechnological products containing EVs. Despite some of the co-isolated impurities investigated in this study not being detectable with the elaborated method, this issue is presumably solvable with the use of staining techniques.

It is worth emphasizing that the number of documented and potential impurities of EVs is significant. However, assuming that the difference in electrophoretic mobility between the impurities and EVs is achievable, the CE technique is expected to provide satisfactory selectivity even without sophisticated detection methods. This makes the CE a complementary tool to currently used assays for the characterization of EVs.

## Data Availability

The data that support the findings of this study are available from the corresponding author (S.D.), upon request.

## Supplementary Materials

The supporting information can be downloaded at: https://www.mdpi.com/article/10.3390/ijms23084347/s1.
