# Supplementary material for: Quality Control of Bacterial Extracellular Vesicles with Total Protein Content Assay, Nanoparticles Tracking Analysis, and Capillary Electrophoresis
Source: Int J Mol Sci. 2022 Apr 14;23(8):4347. doi: 10.3390/ijms23084347 (PMC9028362; doi:10.3390/ijms23084347)
Supplement: Supplementary file 1 [file ijms-23-04347-s001.zip › ijms-1675245-supplementary.pdf]

International Journal of *Molecular Sciences*

MDPI

Article

Quality Control of Bacterial Extracellular Vesicles with Total Protein Content Assay, Nanoparticles Tracking Analysis, and Capillary Electrophoresis

Aleksandra Steć [1], Joanna Jońca [2], Krzysztof Waleron [2], Małgorzata Waleron [3], Agata Płoska [4], Leszek Kalinowski [4,5], Bartosz Wielgomas [1] and Szymon Dziomba [1,*]

1 Department of Toxicology, Faculty of Pharmacy, Medical University of Gdansk, 107 Hallera Street, 80-416 Gdansk, Poland; aleksandra.stec@gumed.edu.pl (A.S.); bartosz.wielgomas@gumed.edu.pl (B.W.)
2 Department of Pharmaceutical Microbiology, Faculty of Pharmacy, Medical University of Gdansk, 107 Hallera Street, 80-416 Gdansk, Poland; joanna.jonca@gumed.edu.pl (J.J.); krzysztof.waleron@gumed.edu.pl (K.W.)
3 Laboratory of Plant Protection and Biotechnology, Intercollegiate Faculty of Biotechnology UG and MUG, 58 Abrahama Street, 80-307 Gdansk, Poland; malgorzata.waleron@biotech.ug.edu.pl
4 Department of Medical Laboratory Diagnostics—Fahrenheit Biobank BBMRI.pl, Faculty of Pharmacy, Medical University of Gdansk, 7 Debinki Street, 80-211 Gdansk, Poland; szeffler@gumed.edu.pl (A.P.); leszek.kalinowski@gumed.edu.pl (L.K.)
5 BioTechMed Centre, Department of Mechanics of Materials and Structures, Gdansk University of Technology, Narutowicza Street 11/12, 80-233 Gdańsk, Poland
* Correspondence: szymon.dziomba@gumed.edu.pl

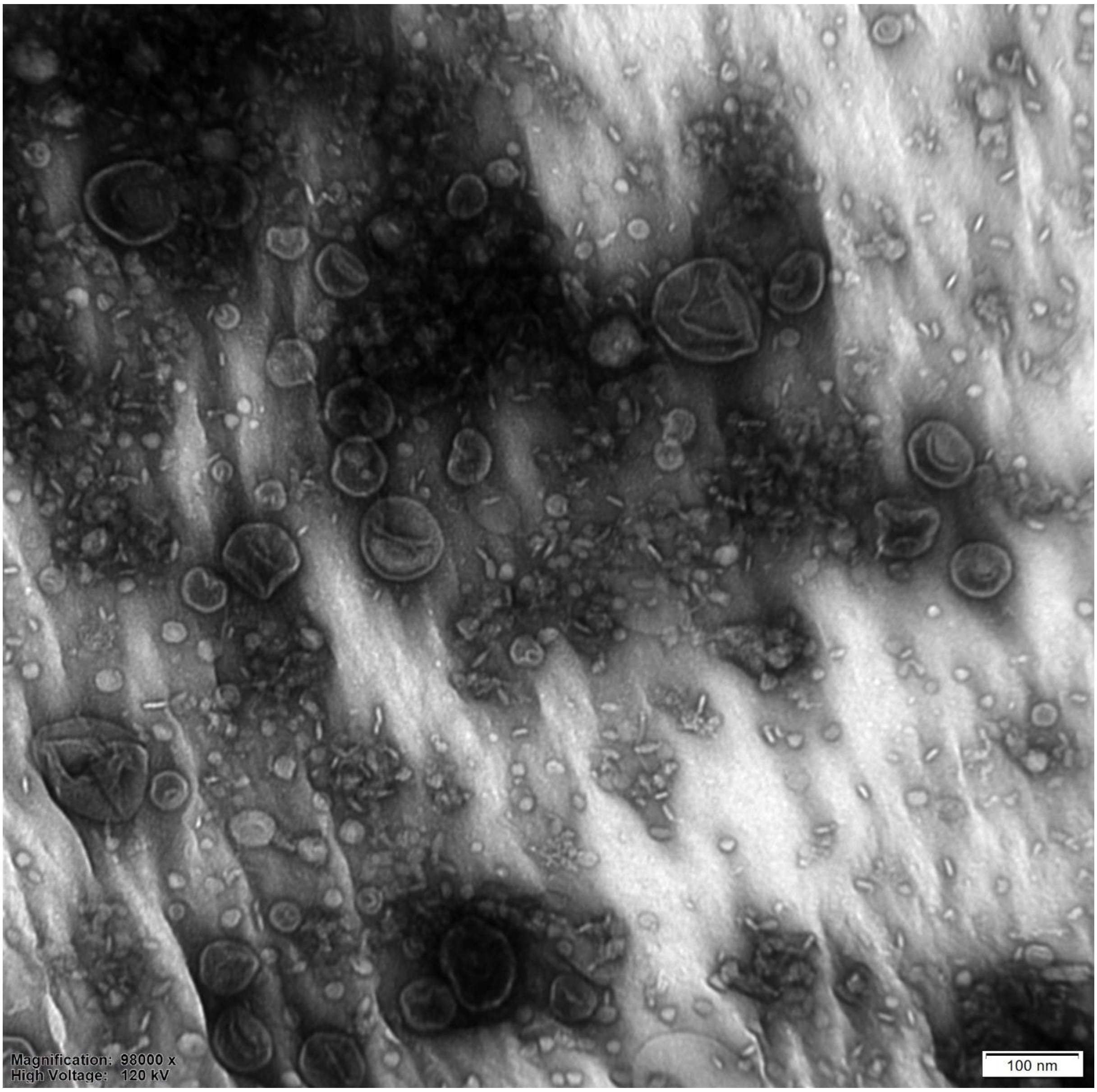

Figure S1. The TEM image of the isolate obtained from *Pectobacterium zantedeschiae* 9M culture using direct UC.

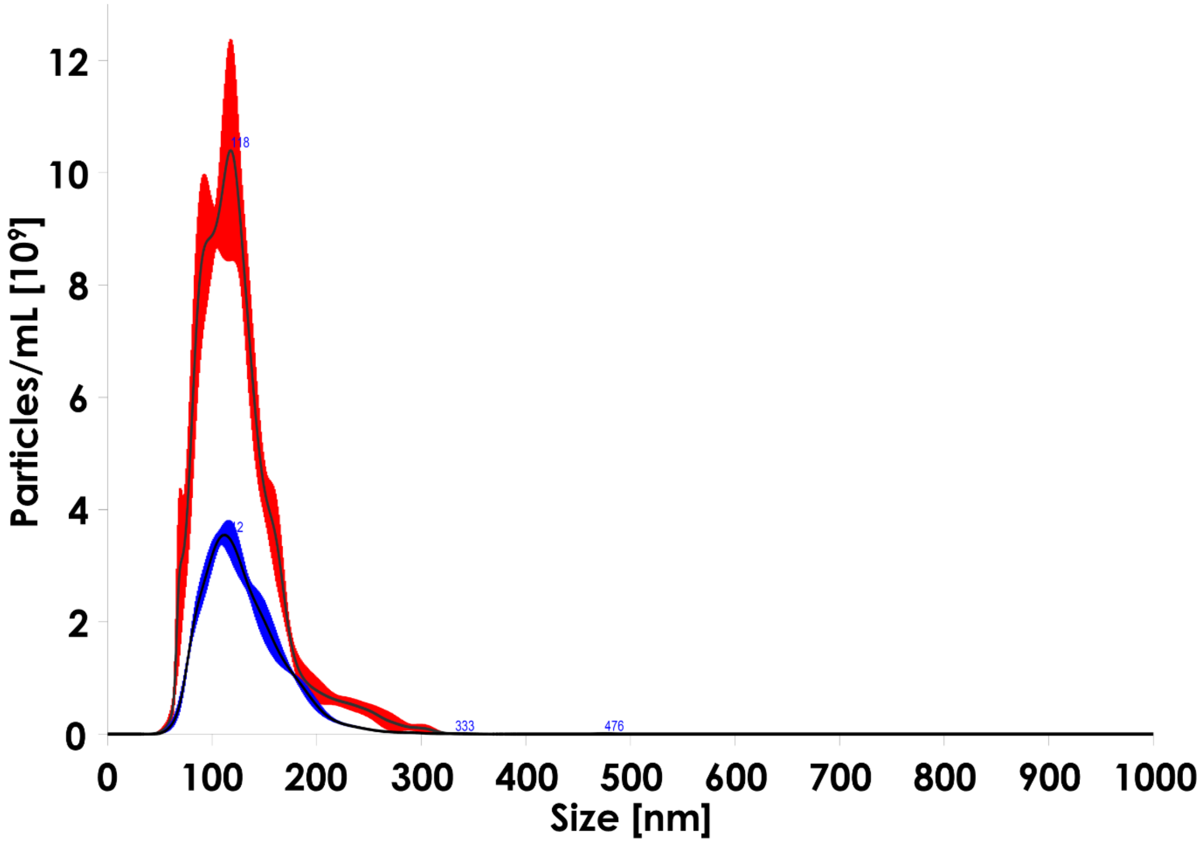

Figure S2. The NTA of isolates of EVs obtained from *Pectobacterium sp.* using (blue trace) direct UC and (red trace) ICUC method.

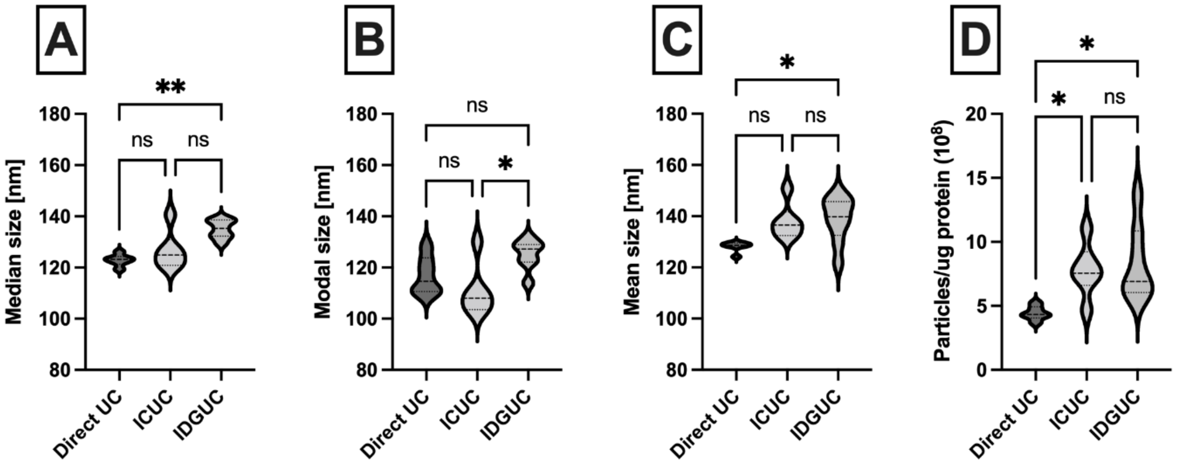

Figure S3. Effect of isolation protocols on (A) median, (B) modal, (C) mean size, and (D) particles/µg protein ratio in isolates of EVs of *Pectobacterium sp*. Kruskal-Wallis test, * p<0.05, ** p<0.01.

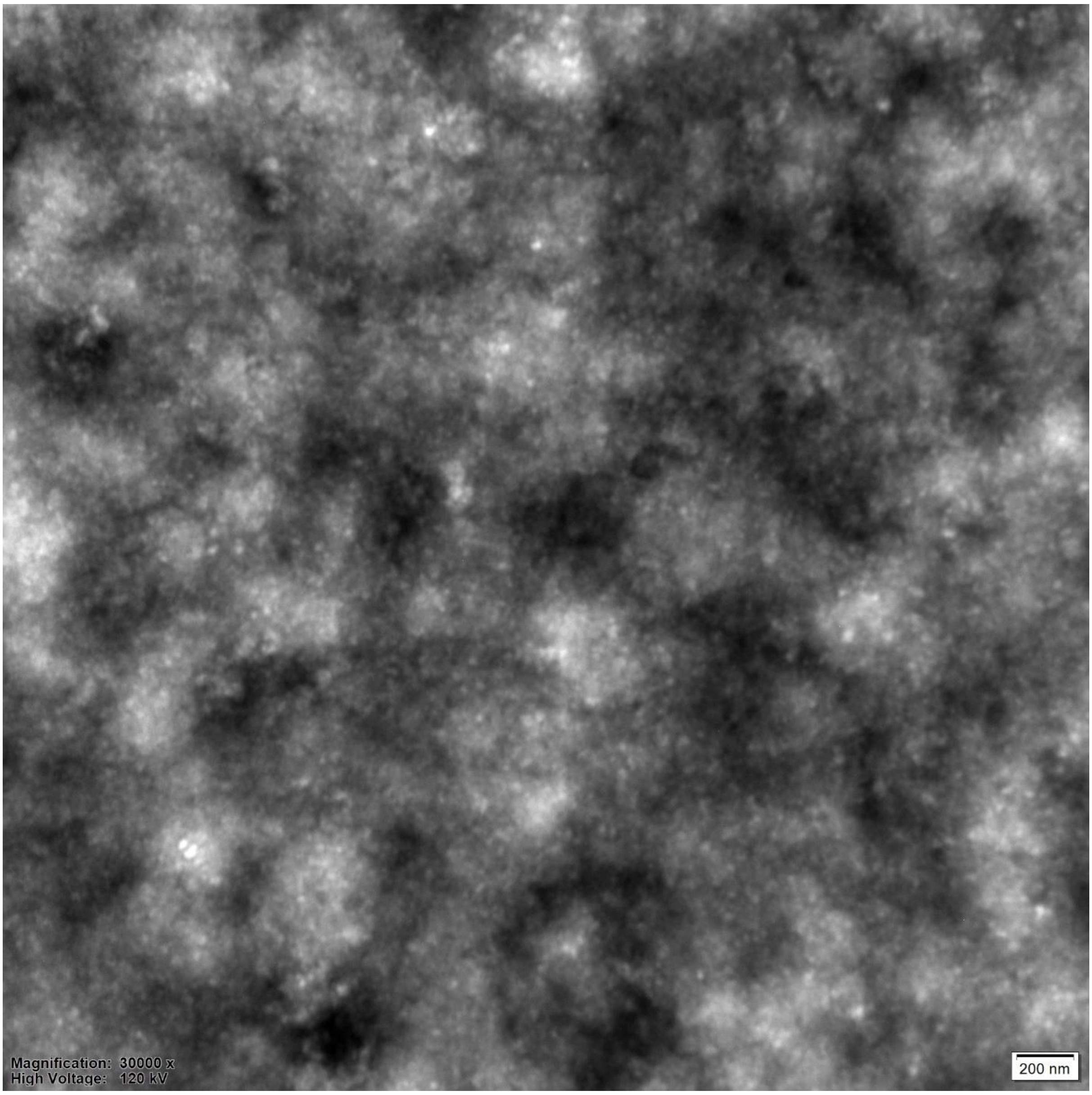

Figure S4. The TEM image of the isolate obtained from M63 medium supplemented with 0.2% glycerol and 0.4% PGA (no vesicular structures were observed).

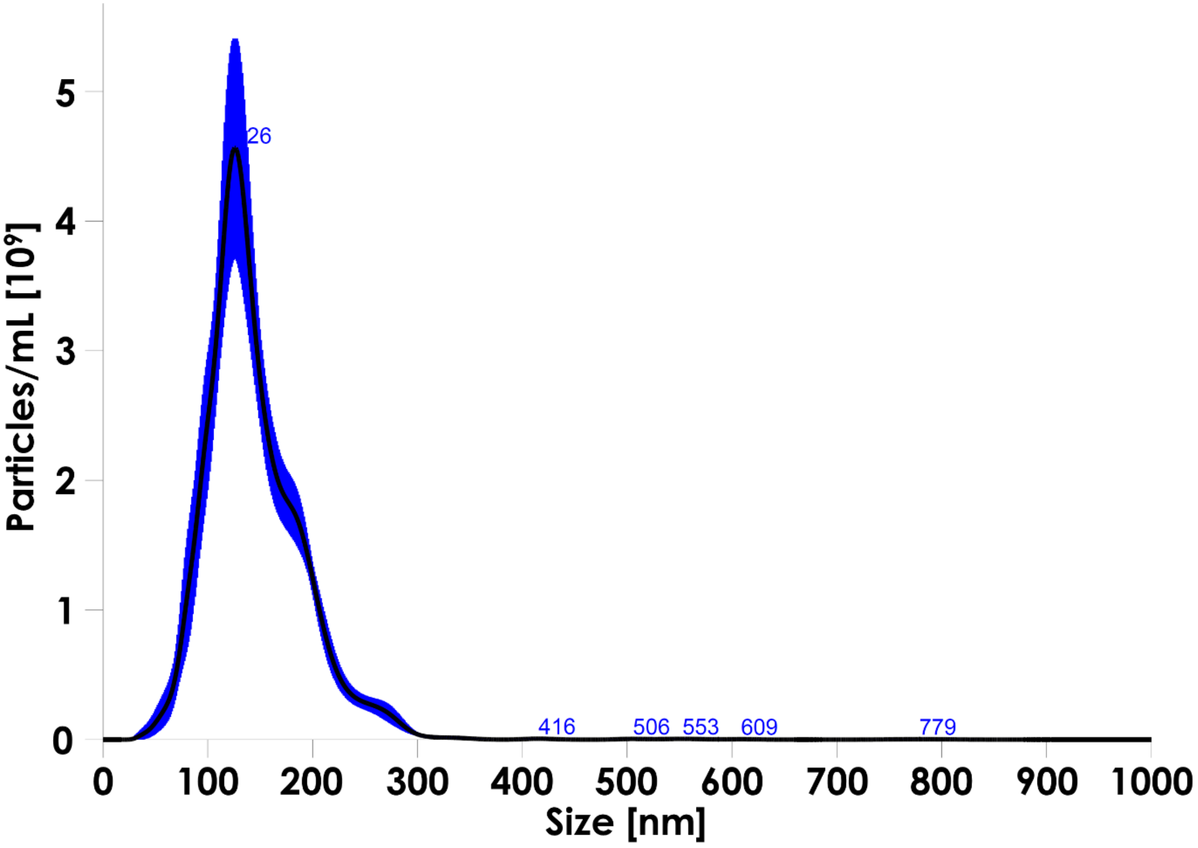

Figure S5. The NTA of the isolate of EVs obtained from *Pectobacterium sp.* using the IDGUC method (Fraction 3).

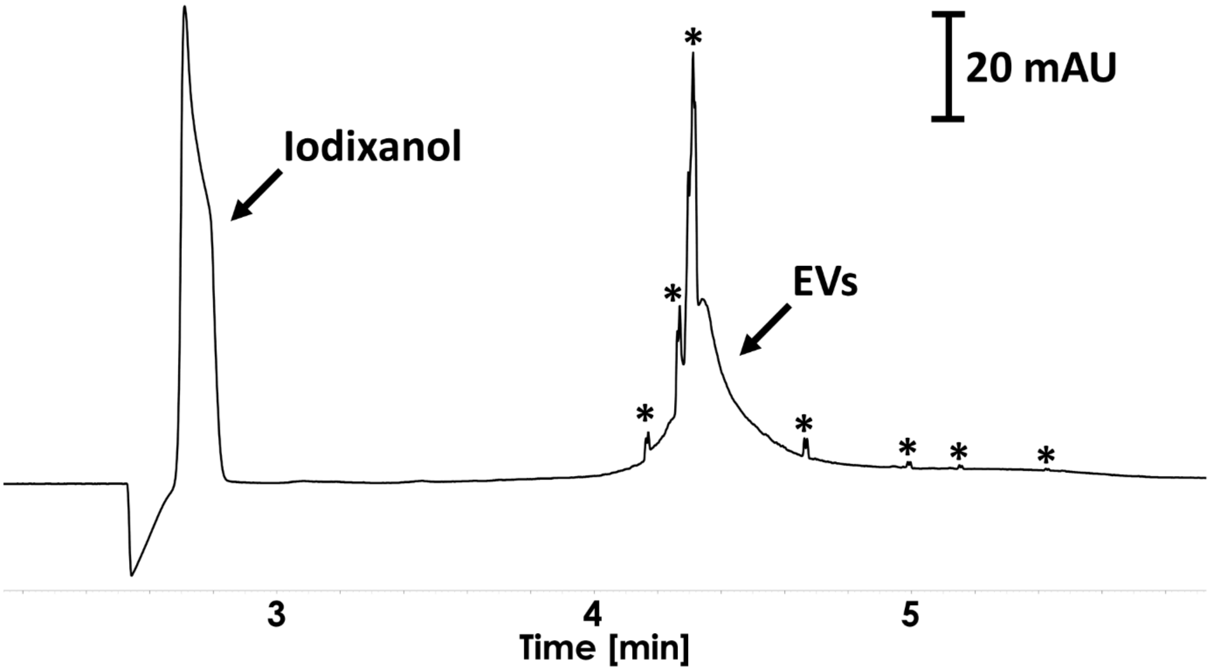

Figure S6. The exemplary CE analysis of low-quality isolate of EVs obtained from *Pectobacterium odoriferum* Car1 culturing medium with ICUC protocol. Asterisks indicate impurities.

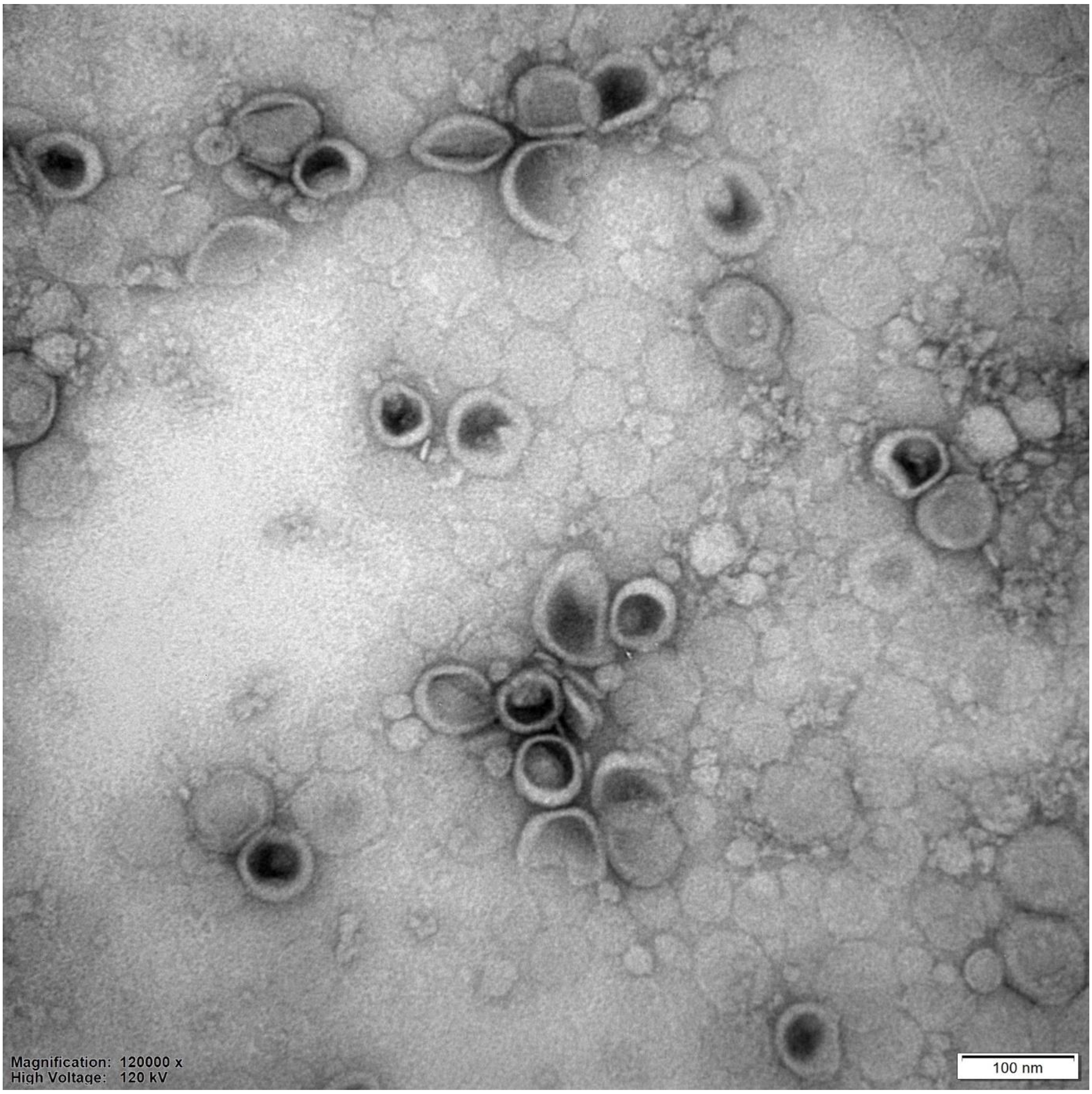

Figure S7. The TEM image of the 9th fraction isolated with the IDGUC method.
